# HX009, a novel BsAb dual targeting PD1 x CD47, demonstrates potent anti-lymphoma activity in preclinical models

**DOI:** 10.1038/s41598-023-32547-y

**Published:** 2023-04-03

**Authors:** Hang Ke, Faming Zhang, Jingjing Wang, Lingxin Xiong, Xiaoyu An, Xiaolong Tu, Cen Chen, Yueying Wang, Binchen Mao, Sheng Guo, Cunxiang Ju, Xiangfei He, Ruilin Sun, Lei Zhang, Owen A. O’Connor, Qi-Xiang Li

**Affiliations:** 1 Hanx Pharmaceuticals, Inc., Hangzhou, China; 2 Crown Bioscience, Inc., San Diego, USA; 3 GemPhamatech, Co., Ltd., Nanjing, China; 4 Shanghai Model Organisms Center, Inc. (SMOC), Shanghai, China; 5 Division of Hematology and Oncology, University of Virginia Cancer Center, University of Virginia, Charlottesville, USA

**Keywords:** Cancer, Drug discovery, Immunology, Biomarkers, Oncology

## Abstract

Both PD1/PD-L1 and CD47 blockades have demonstrated limited activity in most subtypes of NHL save NK/T-cell lymphoma. The hemotoxicity with anti-CD47 agents in the clinic has been speculated to account for their limitations. Herein we describe a first-in-class and rationally designed bispecific antibody (BsAb), HX009, targeting PD1 and CD47 but with weakened CD47 binding, which selectively hones the BsAb for tumor microenvironment through PD1 interaction, potentially reducing toxicity. In vitro characterization confirmed: (1) Both receptor binding/ligand blockade, with lowered CD47 affinity; (2) functional PD1/CD47 blockades by reporter assays; (3) T-cell activation in Staphylococcal-enterotoxin-B-pretreated PBMC and mixed-lymphocyte-reaction. In vivo modeling demonstrated antitumor activity in Raji-B and Karpass-229-T xenograft lymphomas. In the humanized mouse syngeneic A20 B-lymphoma (huCD47-A20) HuGEMM model, which has quadruple knocked-in hPD1xhPD-L1xhCD47xhSIRPα genes and an intact autologous immune-system, a contribution of effect is demonstrated for each targeted biologic (HX008 targeting PD1 and SIRPα-Fc targeting CD47), which is clearly augmented by the dual targeting with HX009. Lastly, the expression of the immune-checkpoints PD-L1/L2 and CD47 seemed co-regulated among a panel of lymphoma-derived-xenografts, where HX009 maybe more effective in those with upregulated CD47. Our data warrants HX009’s further clinical development for treating NHLs.

## Introduction

Lymphomas are a heterogeneous group of malignances comprised of both highly aggressive and indolent diseases. In general, the WHO Classification of Hematopoietic Tumors recognizes nearly 80 discrete subtypes of non-Hodgkin’s lymphoma (NHL), with an annual incidence of about 80,000 cases in the U.S. The emergence of biological therapies, as first shown with rituximab in 1998, have transformed the management of these complex diseases. Presently, a diverse spectrum of biological agents is used to treat these diseases, including monoclonal antibodies (mAb) (targeting CD20, PD1/PDL1), antibody drug conjugates (ADC) (Brentuximab vedotin, Polatuzumab vedotin), bispecific antibodies (BsAb) (blinatumomab) and CAR-T. While each treatment modality has exhibited marked activity in defined contexts, the activity seen with any one has been relatively restricted, with often less than promising activity across the majority of other NHL subtypes. Evolution of more recent treatment platforms is beginning to gradually integrate traditional chemotherapy and precision targeted drugs with different biologics, transforming the standard of care (SOC) for many subtypes of NHL in a relatively short timeframe.

PD1 and its ligands PD-L1/2 constitute an important pathway modulating host T-cell activity and antitumor cell mediated immunity^1,2^. In the case of lymphoma, the approval of PD1 targeted drugs including pembrolizumab and nivolumab for the treatment of Hodgkin’s Lymphoma (HL) and nasal NK-T cell lymphomas has represented an incremental improvement in these diseases, though the activity of these checkpoint inhibitors has not been seen across the greater diversity of NHL^3–5^.

Similarly, CD47, a “don’t eat me” receptor, is over-expressed in many human tumors, including hematological malignances^6–12^. CD47 and its ligand, signal regulatory protein α (SIRPα, residing on the surface of phagocytic cells, including macrophage and dendric cells, or DCs), constitutes a key innate, as well as adaptive, immune checkpoint^13,14^. High expression of CD47 in tumor cells has frequently been associated with poorer prognosis across many types of cancers^12,15,16^. Together, these proteins have been considered important immune-checkpoint targets for cancer therapy^6,14,17,18^, including aggressive lymphomas like diffuse large B-cell lymphoma^8^, and other hematological malignances like cutaneous T-cell lymphoma^10,19,20^. To date, targeting CD47 has been achieved with an investigational anti-CD47 mAb drug (e.g. magrolimab, also called Hu5F9-G4)^8^, and with a SIRPα fusion protein^21,22^, which blocks the CD47-SIRPα interaction. While both have demonstrated encouraging activity against a variety of hematological malignances, including lymphoma^8,10,19,20,23^, neither has been approved to date for any indication. The major challenges in the clinic have been largely attributed to the modest single agent antitumor activity^24,25^, and their association with a variety of dose limiting toxicities (DLT). Anemia and thrombocytopenia, common with anti-CD47 targeted therapies, are attributed to the broad CD47 expression on megakaryocytes and erythrocytes^25^. In theory, designing unique features of CD47 targeted therapies could circumvent some of these toxicities, and leverage any synergistic interaction that may exist with these two classes of checkpoint inhibitors.

A number of approaches have been considered to improve the activity of these agents. The first would be to create an ADC by conjugating the CD47 targeted antibody to a warhead like monomethylauristatin E (MMAE), as done with BV^24^. The second would be to design rational bispecific antibodies which might leverage a favorable drug:drug interaction that could augment the activity of the combination above that of the monotherapy^13,26^. Such an approach would allow one to engage aspects of both the innate and adaptive immune response^13,27–29^. The third consideration would be to engineer differential affinity for the alternative non-CD47 targeted component of the bispecific, allowing for greater binding interactions which should reduce binding to off-tumor CD47^29,30^.

Development of new drugs including immunotherapies relies heavily on the use of experimental models that allow for quantitating anti-tumor effect in the proper biological context. While no one model is absolutely predictive of clinical outcome, each has its own strengths and limitations, and can provide important insights into the drug mechanism of actions (MOAs)^31^. Herein, we report on the complimentary interactions of a rationally designed and dual targeted BsAb (CD47 x PD1), HX-009, which appears to exhibit superior activity over the monotherapies in various preclinical models of B- and T-cell lymphomas.

## Results

### Construct a new BsAb (CD47 × PD1), HX009

**Figure 1:**
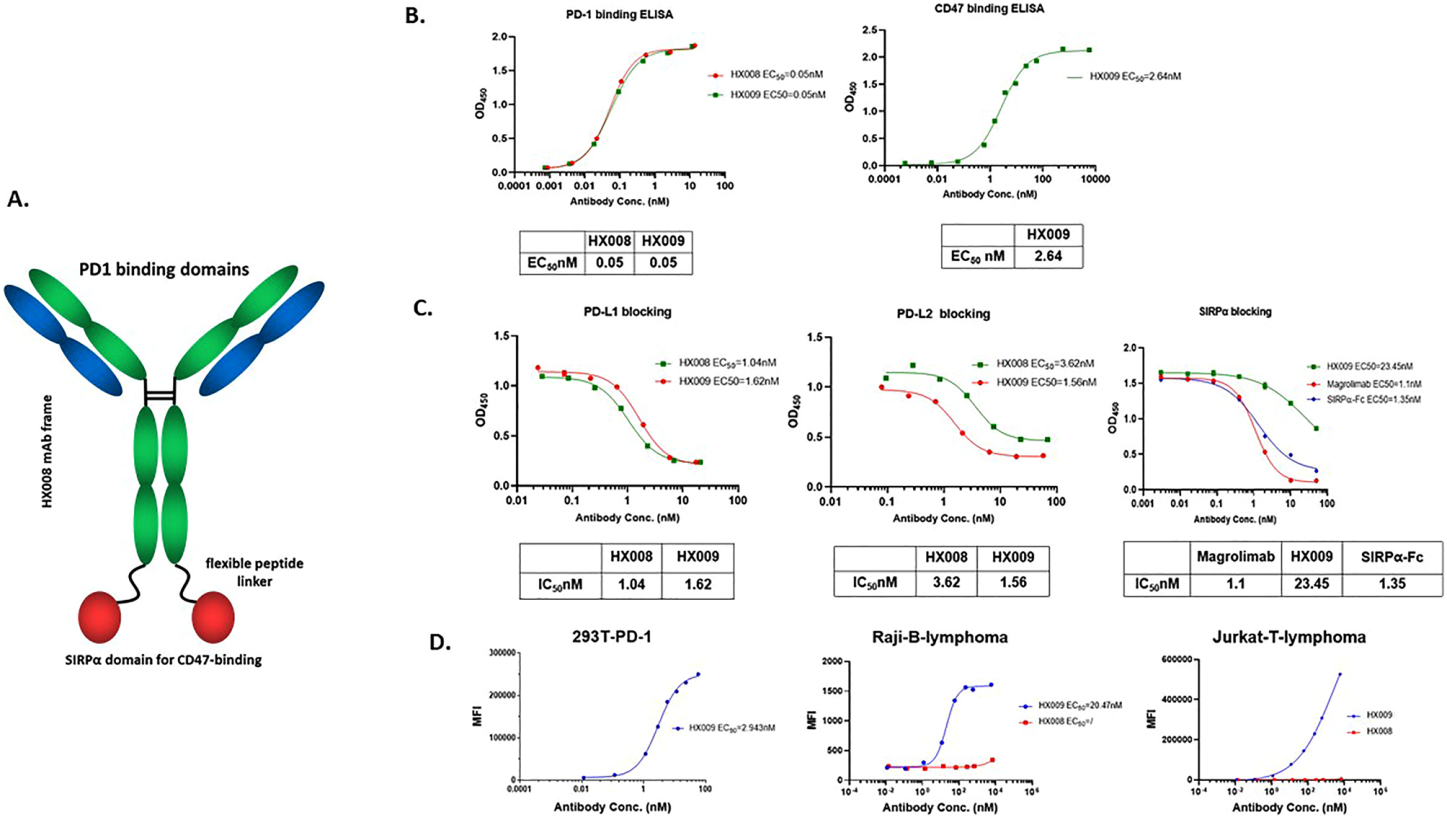
In vitro biochemical characterization of HX009. (**A**) Molecular structure of HX009: 2 × 2 symmetric BsAb molecule; (**B**) HX009 binding to PD1-his and CD47-his recombinant proteins as measured by ELISA assay; (**C**) HX009 competitively blocks PD1 and CD47 receptor binding to their respective recombinant ligand proteins, PD-L1-his, PD-L2-mFc and SIRPα-mFc; (**D**) HX009 binding to PD1-expressing 293 cell lines in vitro as measured by flow cytometry (Left panel), and to the CD47^+^ lymphoma cell lines (Raji B- and Jurkat T-lymphoma cells, presented by mean fluorescence intensity (MFI))(Middle and right panels). IC_50_ (half maximal inhibition concentration) and binding EC_50_ (half maximal effective concentration) were determined per four-parameter equation fitting curves.

Dual targeting of both innate and adaptive checkpoints is theoretically attractive, also since data suggest that dual targeting of discrete checkpoints, e.g. CD47, PD1/PD-L1 or CTLA4, can be synergistic with regard to their anti-tumor effects^13,27,29^. We set out to create a BsAb targeting both human PD1 and CD47 in order to create a novel biologic with enhanced anti-tumor activity and reduced hemotoxicity based on rational design. We previously developed, and now approved, a humanized IgG4 PD1 mAb, HX008, which demonstrated strong antitumor activity across a variety of tumor types, a stable biochemical structure and a highly favorable pharmacokinetic property^32–35^. We used its molecular frame to create a new BsAb, HX009, by grafting the extracellular CD47-binding domain of human SIRPα protein at the C-terminus (Fc) of the heavy chain of HX008 as shown in Fig. 1A (The detailed construction will be published separately). The “2 × 2” symmetric format ensures that the high affinity PD1 binding is maintained while the SIRPα domain has significantly reduced CD47 binding affinity, aiming at reducing binding to CD47^+^ RBCs and megakaryocytes to minimize hemotoxicity (data not shown here), and also to reduce the anti-CD47 “antigen-sink” effect due to the broad expression of CD47 among normal tissues. PD1 has little systemic expression, but is expressed in tumor microenvironment (TME), so HX009 has tumor targeting effect^30^, helping the weakened SIRPα binding to CD47 on tumors. The detailed information of HX009 molecule, its production and the finished drug product (DP) as an IV injectable solution (10 mL/vial and 100 mg/vial) used in various preclinical investigations are separately described elsewhere.

### Confirmation of HX009’s strong binding to PD1 and less potent binding to CD47

The primary feature of HX009 is its ability to bind and block pre-determined targets of interest. Figure 1B shows the binding, as measured by ELISA assay, confirming the binding interactions of HX009 to recombinant PD1-his and CD47-his proteins. HX009 exhibited identical specific binding to PD1 protein as the parental HX008 molecule, with an EC_50_ ~ 0.05 nM, confirming the maintained PD1 binding affinity with the addition of SIRPα domain at its Fc domain. HX009 also binds to CD47 with an EC_50_ of approximately 2.6 nM, which is about 50-times less potent than that observed with the anti-PD1 component. This is also in contrast to the binding affinity of Fc-SIRPα (EC_50_ ~ 0.24 nM), and magrolimab (Hu5F9-G4, EC_50_ ~ 0.085 nM in our hand, data not shown)^8^. These data underscore the intended HX009 design features, namely that the CD47 affinity is reduced compared to the PD1 targeted component (IC_50_ ratio ~ 52 × between PD1 and CD47), and in contrast to the molecule presently under study in the clinic.

Beside binding to the receptors, we sought to quantitate the blocking potential of HX009 against its target receptors’ binding to their respective ligands, recombinant PD-L1mFc, PD-L2-his and SIRPα-mFc, using a competitive ELISA assay. Figure 1C demonstrates that the PD-L1 blocking dose–response curve (IC_50 ~_1.6 nM) of HX009 are only slightly lower than that for HX008 (IC_50_ ~ 1.0 nM), while the PD-L2 binding blocking dose–response curve of HX009 (IC_50_ ~ 1.6 nM) is slightly greater than that of HX008 (IC_50_ ~ 3.62 nM). The SIRPα-mFc blocking IC_50_ of HX009 was shown to be approximately 23.4 nM, in contrast to the blocking dose–response curve of the native SIRPα-Fc with IC_50_ of approximately 1.4 nM and that of magrolimab with IC_50_ of approximately 1.1 nM^8^.

We also assessed HX009 binding to PD1^+^ cell line, engineered 293T cell over-expressing PD1, by flow cytometry measuring both mean fluorescence intensity (MFI) and/or % positive cells. The dose–response curve, as shown in Fig. 1D, demonstrates that HX009 specifically binds this PD1^+^ cell line with EC_50_ of approximately 2.9 nM. We then went on to confirm HX009 binding to selected CD47^+^ lymphoma cells (Supplement Fig. S1 showing mRNA levels), Raji B- and Jurkat T-lymphoma lines by flow cytometry with the binding dose–response curves shown in Fig. 1D (EC_50_ 21 nM for Raji), in contrast to the minimal binding seen with HX008.

### In vitro functional characterization of HX009 using reporter cell lines

**Figure 2:**
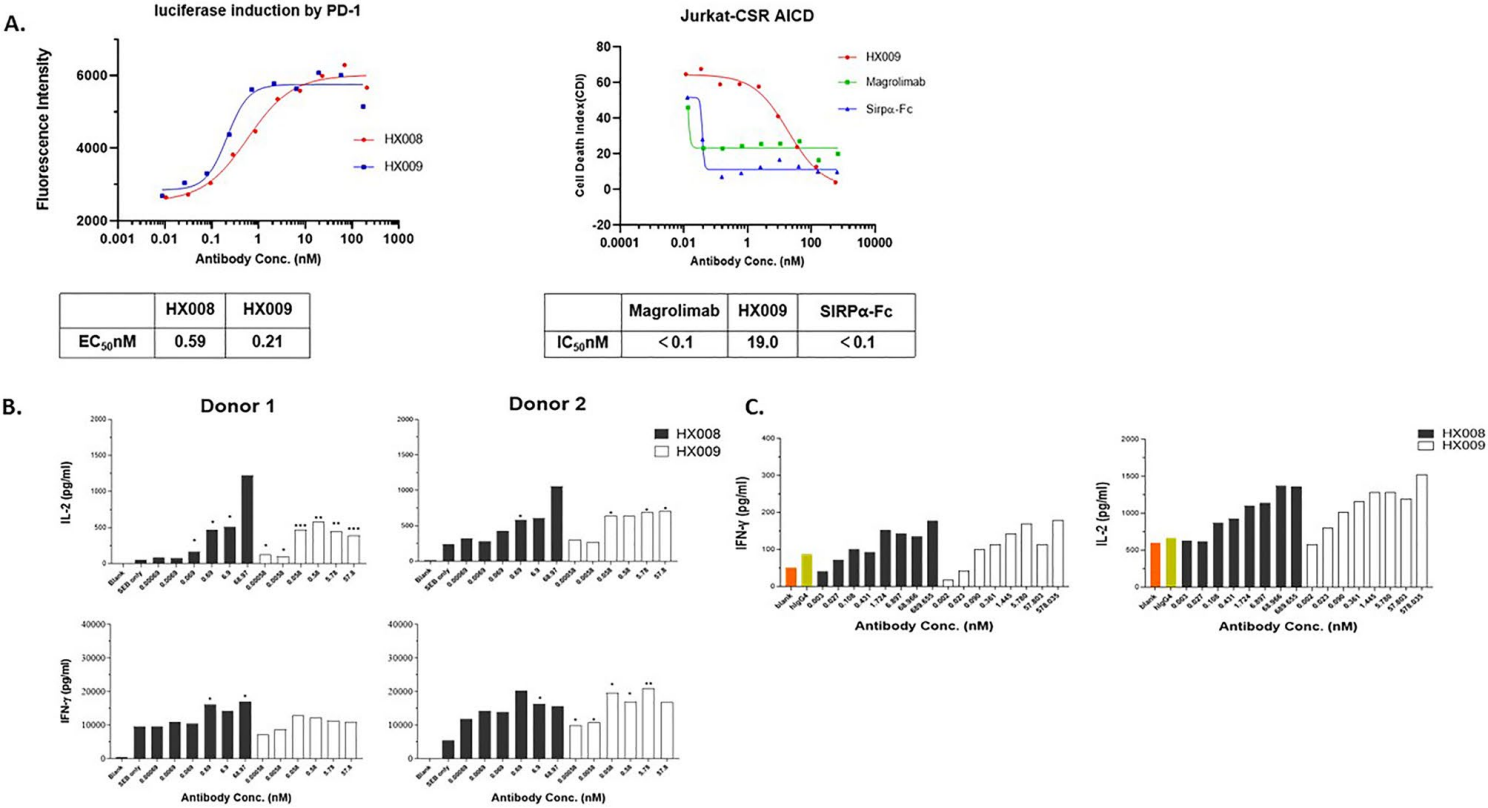
In vitro biological function characterizations of HX009. (**A**) Reporter assays for both target engagement. Left panel: Different concentration of HX009 and HX008 activated luciferase reporter expression in reporter cell lines by down-regulating PD1 /PD-L1 interaction, and EC_50_ was determined according to four-parameter equation fitting curves^36^; Right panel: CD47-hFc induced activation induced cell-death (AICD) in Jurkat-CSR reporter cells, where reporter cells were incubated with different concentrations of HX009, Magrolimab and hSIRPα-Fc to block CD47-SIRPα interaction to prevent reporter cells from AICD; IC_50_ was calculated per the four-parameter equation fitting curves. Data were shown as average signal, n = 2. (**B**) T cell from two different donors were SEB stimulated, followed by reactivation by HX008 and HX009 after. IL-2 (upper panel) and IFN-γ (bottom panel) secretion were measured via an ELISA method. SEB only treatment was also measured for basal cytokine level. Data were shown as average signal, n = 2. *p*-values: ***:0–0.001, **: 0.001–0.01, *: 0.01–0.05 compared to the SEB-only treatment. (**C**) Activation of lymphocytes by HX009 treatment in mixed lymphocyte reaction (MLR) assay. IL-2 (left panel) and IFN-γ (right panel) secretion were measured via an ELISA method. Blanks and IgG4 isotype control treatment were also measured for basal cytokine level. Data were shown as average signal, n = 2.

Following confirming biochemical binding/blocking, we sought to determine the HX009 binding’s impact on signaling in the respective biological pathways. While the biological function of anti-PD1 is likely maintained per design, confirming the biological function of the CD47 fragment is critical given the lower binding affinity. One of the approaches commonly adopted to understand functional interactions between ligands and receptors is to utilize a reporter system where the reporter readouts (*e.g*. luciferase activity) represent a surrogate function readout of ligand-receptor interaction. With that, we first tested HX009 in a reporter assay for PD1 blocking function, where Jurkat cell line was engineered to carry an IL-2 promoter-guided luciferase reporter gene^36^, and the luciferase signal mimics the T cell activation by blocking PD1/PD-L1 interaction, when this line is cocultured with a CHO cells expressing TCR stimulatory molecules (an OKT3 clone anti-CD3 single chain antibody fragment (ScFv)) and PD-L1. We confirmed and quantified the biological activity of HX009 PD1 blockade as shown in Fig. 2A using this assay. Interestingly, we have also noticed consistent higher functional readout (nearly 3 × EC_50_) for HX009 over HX008, in contrast to the same or even slightly lower binding/blockae as compared to HX008 (Fig. 1A,B). If this is true, it is possible that the CD47 engagement in T-cells by HX009 could potentially enhance the T-cell activation pathway by PD1 blockade as suggested by others recently^37^, and that HX009 maybe more effective as an ICI than PD1 mAb (*e.g.* HX008). All these would need to be confirmed with further experimentations.

Similarly, we also assessed the CD47 function blockade by HX009 using another specially designed reporter assay. In this assay, a Jurkat cell line (also called “Jurkat-CSR”) stably transfected with a chimeric-SIRPα-receptor (CSR) composed of CD47-binding domain of SIRPα, transmembrane domain of CD28 and the intracellular CD3ζ signal transducing domain. CD47 gene was removed from on Jurkat-CSR cell via gene knock-out (KO) to avoid self-binding within cell (https://patentscope2.wipo.int/search/en/detail.jsf?docId=CN297701761&_cid=JP1-LBPTA7-89214-1). When ligated with CD47, the cell triggers strong CD3 activation signal mediated cell death (AICD), which can be prevented by the anti-CD47 antibody or soluble SIRPα mediated neutralization. We, using this cell, confirmed the functional blockade of CD47 by HX009 as shown in Fig. 2A, although with lower potency as compared to magrolimab and SIRPα-Fc control.

### In vitro assessment of T-cell activation by HX009 ant-PD1 function

To address the functional aspects of the PD1 component, we next assessed the T-cell activation function by PD1 blockade in two commonly used in vitro assays. First, we introduced HX009 to the peripheral blood mononuclear cells (PBMC) pretreated with SEB (Staphylococcal enterotoxin B). As a super-antigen, SEB activates T-cells at low concentration and for a long time, rendering them exhausted. The exhausted T-cells can be reactivated by anti-PD1 antibody and produce IL-2/IFN-γ that can be measured as readouts for anti-PD1 functions. Using this assay and two donors’ human PBMC, our results revealed that HX009 can reactivate the exhausted T-cells (Fig. 2B), similarly as HX008, confirming PD1 functional blockade by HX009. Second, a standard mixed lymphocyte reaction assay (MLR) was used to assess HX009’s ability to enhance T-cell activation during the mix lymphocyte culture by direct measurement of IL-2 and IFN-γ productions. As shown in Fig. 2C, HX009 treatment further enhanced the production of both IL-2 and INF-γ in a concentration-dependent fashion, similar to that of HX008. These results confirmed the PD1 functional blockade and enhancement of T-cell activation by HX009. It worth mentioning that we have not seen apparent high activity for HX009 than for HX008 in these two assays.

### HX009 demonstrated anti-tumor activity in two different lymphoma cell line derived xenograft models

Following the confirmation of the designed properties of HX009 in vitro, we evaluated the anti-lymphoma activity in vivo using various preclinical models with certain predictive power (Table 1)^31^. While there is no one perfect in vivo model to assess these types of immunotherapies, each type of mouse models can however assess certain aspects of drug’s antitumor activity. For instance, xenograft lymphomas can only grow in immunodeficient mice (which have no T-cells), so full assessment of the PD1 targeting cannot be determined. In contrast, xenografts are potentially adequate in assessing the CD47 targeting effect (Table 1). On the other hand, humanized mouse tumor models can also be adopted for evaluating PD1 and CD47 targeting in immune-competent mice (Table 1)^31^. To explore the potential anti-lymphoma effects of HX009, we conducted several sets of in vivo murine studies using multiple types of preclinical lymphoma models to answer specific questions, including: (i) Raji B-lymphoma (Burkitt’s) xenograft; (ii) Karpas-299T-lymphoma (an anaplastic large cell, or ALCL) xenograft, (iii) A20 syngeneic mouse B-lymphoma (homograft)^31^, and (iv) a small cohort of DLBCL PDXs (Table 1).

**Table 1:** Preclinical cancer models used in this study.

Model type	Model	Features	Comments
Xenograft lymphoma	Raji B-lymphoma	Human lymphoma xenograft growth in NOD immune-deficient mice. No PD1 targeting can be assessed, while CD47 targeting can be assessed pharmacologically	Cell-derived xenograft
	Karpas-299 T-lymphoma		
Humanized syngeneic lymphoma (HuGEMM)	huCD47-A20 B-lymphoma syngeneic model	Human PD1, PD-L1, CD47 and SIRPα were knocked-in to immune-competent mice, so both PD1/CD47 targeting can be assessed pharmacologically	Cell-derived homograft
DLBCL PDXs	LY2345, 6701, 12,966, 12,962, 2266, 2264	Similar to other xenografts above, where PD1 targeting cannot be assessed while CD47 targeting can be assessed. Have similar molecular pathology and similar heterogeneity as in patients	A cohort of PDXs can be modeled in clinical trial style trial

**Figure 3:**
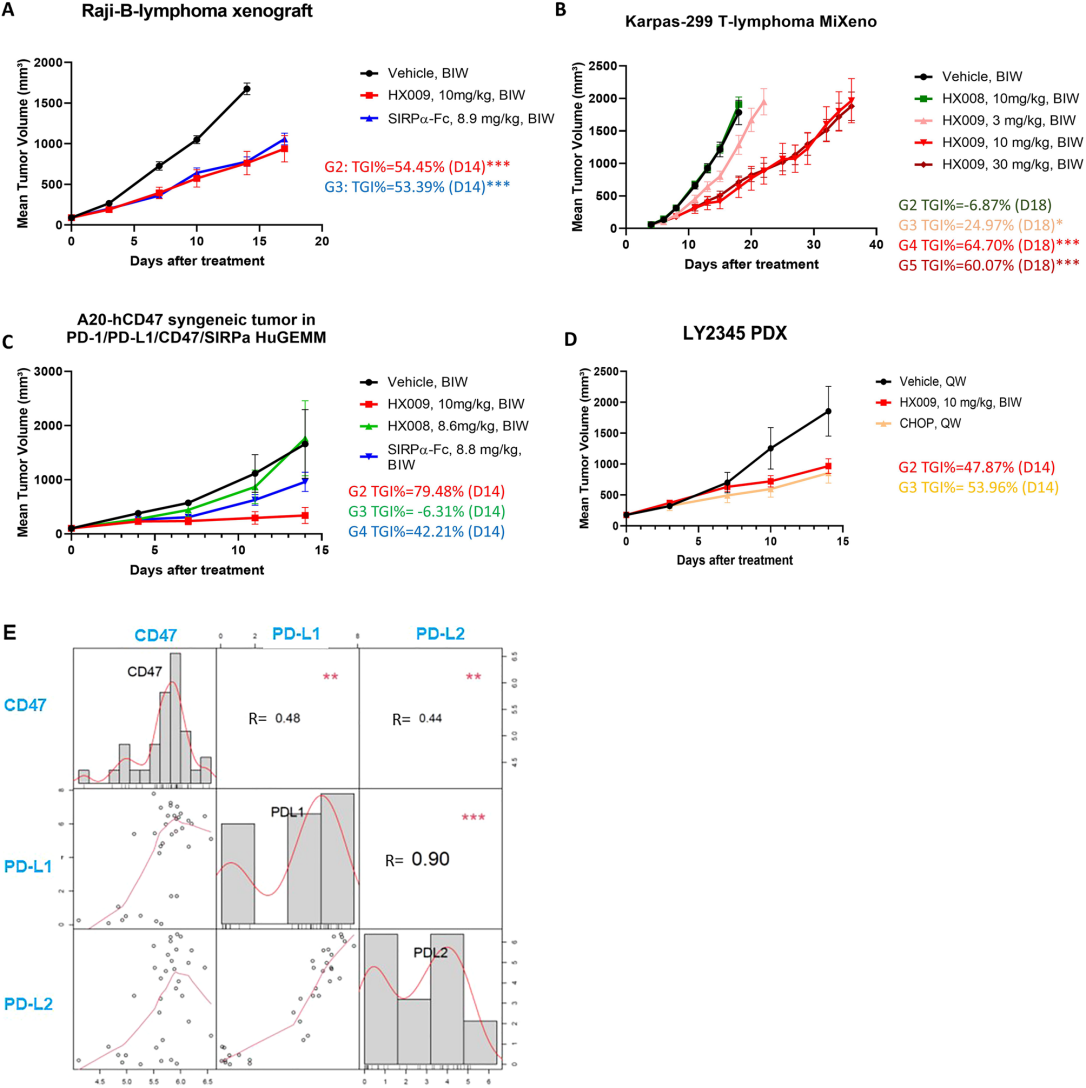
In vivo anti-lymphoma activities HX009 in three different types of preclinical lymphoma models (Table 1) as presented by the tumor growth inhibition curves (**A**–**D**), and the expression correlations among the immune checkpoints, *CD47*, *CD274* (encoding PD-L1) and PD-L2 genes in DLBCL (**E**). Tumor growth was measured twice a week and is shown as average tumor size per group ± SEM. Statistical significance was determined by one-way ANOVA. Gene expression correlations at mRNA levels measured by RNAseq (log2(TPM). TPM represents "Transcripts Per Kilobase Million" and the correlation at protein levels measured by IHC-H-scores. (**A**) Human Raji-B-lymphoma xenograft model (The treatment TGI values were displayed next to the curve; G2: HX009 group and G3: SIRPα-Fc group); (**B**) Human Karpas-299 T-lymphoma xenograft (“MiXeno”) model (G2: HX008 group, G3: HX009-3 mg/kg group, G4: HX009-10 mg/kg group, G5: HX009-30 mg/kg group); (**C**) Humanized mouse syngeneic A20-B lymphoma model (HuGEMM PD-L1 × PD1 × CD47 × SIRPα) (G2: HX009 group and G3: HX008 group, and G4: SIRPα-Fc group); (**D**) A representative lymphoma PDX (G2: HX009 group and G3: CHOP group); (**E**) Correlation of the CD47, PD-L1 and PD-L2 mRNAs levels among the DLBCL-PDXs, the corresponding R-values/p-values are displayed in the figure, *p*-values: ***:0–0.001, **: 0.001–0.01, *: 0.01–0.05.

The principle basis for testing biologicals targeting human CD47 using xenograft tumor models revolves around the fact that the polymorphic SIRPα in NOD mice interacts with human CD47, supporting the growth of xenografts^38,39^. In the first study of a subcutaneous Raji xenograft lymphoma model^26^ in immuno-compromised NOD/SCID mice, HX009 (10 mg/kg) induced significant growth delay (approximately 54% TGI, or Tumor growth inhibition = (1 − V_treatment_/V_control_) × 100), similar to SIRPα-Fc (53% TGI) (Fig. 3A), confirming the anti-lymphoma effects of a CD47 directed biologic in this immunocompromised xenograft model. In this CD47^+^ xenograft model (Supplement Fig. S1), the absence of a functional human lymphoid immune system, coupled with identical dose response curves for HX009 and SIRPα-FC, suggests the activity seen was entirely attributed to the anti-CD47 effect, without any contribution from the anti-PD1 component of HX009. Furthermore, the observed anti-lymphoma activity in this xenograft study, along with those observed with the following lymphoma xenograft pharmacology studies, confirmed that direct targeting tumor cell CD47 by HX009 resulted in anti-lymphoma effects without targeting CD47 on TME (*e.g.* those on immune cell, vascular cells, stromal cells, etc.) since the TME belongs to mouse components.

In the second model, Karpas-299 (a T-cell lymphoma) was co-transplanted with human PBMC (Mixeno™)^31,40^, with the intent to measure the PD1 blockade effect beyond just CD47 blockade as in Raji modeling. HX009 induced remissions in a dose dependent fashion in this model (Fig. 3B), with statistical significance at both high dose levels (10 and 30 mg/kg, TGI: 60–64%). However, HX008 showed little anti-tumor effect, likely due to lack of an autologous anti-tumor immune system in this model^31^. That is, the observed response to HX009 was entirely attributed to the anti-CD47 effect in this scenario. Together, these data confirmed the anti-lymphoma activity of HX009 across both the CD47^+^ B- and T-cell lymphomas (Supplement Fig. S1), due to the contribution of the SIRPα fragment in HX009.

### HX009 exhibits augmented anti-lymphoma activity in humanized syngeneic mouse lymphoma model, superior to the respective single targeting agents

Given the absence of a competent autologous immune system in conventional xenograft models, including MiXeno humanized mice as for above Karpas-299 model^31^, it is not possible to assess the activity of PD1 blocking effects of HX009. An alternative type of humanization is to substitute mouse gene targets by human counterparts via genetic engineering (knock in), both in syngeneic tumor cells (“HuCell”) and in mice (“HuGEMM”)^31^, creating a ‘new’ humanized mouse model. Recently, these models have been employed to understand the antitumor effects of biological therapeutic agents intended for human use^31,41–43^ (Table 1). To this end, we constructed a quadruple human gene knock-in (KI) Balb/c mice: PD1, PDL-1, CD47 and SIRPα (HuGEMM-PD1xPDL-1xCD47xSIRPα), similar to those used by others^44^ and a human CD47-KI mouse syngeneic A20 B-cell lymphoma, as schematic described in Supplement Fig. S2. This humanized syngeneic mouse B-lymphoma A20 model contains autologous tumor immunity that can be employed to assess dual targeting MOA simultaneously. Our results demonstrated that while the single agents targeting either PD1 (HX008) or CD47 (SIRPα) have anti-tumor activity, as shown in Fig. 3C, the dual targeting by HX009 induced a synergistic anti-tumor activity, as shown in Fig. 3C. These data, in this complex model system that leverages the need for host innate immunity, strongly supports the notion of enhanced treatment benefit through the dual targeting of both CD47 and PD1. Recently, a new mechanism of anti-PD1 mediated T-cell activation via SIRPα on tumor cells interacting with CD47 on CD8^+^ T_eff_ in TME has been described^37^. It is possible that the observed anti-lymphoma activity from anti-CD47 function of HX009 could be the HX009-SIRPα interaction either with tumor cell CD47 or with the T_eff_ CD47, or both. The present experiment cannot rule out either of the potential MOAs.

### Immune checkpoints (ICs) seemed to be co-regulated in lymphoma-PDXs

With demonstrated anti-lymphoma activities in the above lymphoma cell derived preclinical models, we sought to determine if the same was true in models using patient derived xenograft (PDX^45^). In particular, a panel of PDX models, closely recapitulating specific human diseases and their heterogeneity, was used to evaluate HX009 under these conditions^46–49^. It is reasonable to speculate that the expression levels of the targets of HX009, the ICs PD1 or its ligands, PD-L1/2, and CD47, in lymphoma could be important parameters that influence HX009 efficacy. We first need to know if lymphoma PDXs, by mimicking patients, express these ICs. We recently described establishment and genomic profiling of a panel of lymphoma PDXs (Tables 1 and 2). We thus investigated molecular epidemiology of ICs in this cohort of PDXs, including PD-L1/L2 and CD47 gene expression, either mRNA levels by RNAseq or protein levels by immunohistochemistry (IHC) (CD47 and PD-L1 only, scored by H-scores) (Table 2). The representative images of lymphoma PDX CD47 and PD-L1 IHC are shown in Supplement Fig. S3A, demonstrating varying degree of CD47 protein expressions. Importantly, the H-scores of IHC are not well correlated with mRNA levels of these models by RNAseq for CD47, as per Spearman’s co-efficiency correlation (CC) analysis (Supplement Fig. S3B left panel), although a slight trend is there. In contrast, IHC H-scores using the commercially available Companion diagnostic kit (CDx) are correlated with mRNA levels by RNAseq for PD-L1 with statistical significance (Supplement Fig. S3B right panel). Most interestingly, the three ICs, PD-L1/L2 and CD47 mRNA levels are co-regulated among the cohort of lymphoma PDXs with statistical significance per Spearman’s CC analysis (Fig. 3E)(Table 2), although the underlying mechanisms still need to be elucidated. The similar trend of the co-regulation seems to be seen in DLBCL patients (Supplement Fig. S3C), although short of statistical significance due to small sample size. Altogether, this further supported the rationale of the dual PD1-CD47 targeting for treating lymphoma. IHC or RNAseq assays could be potentially developed as predictive biomarkers for HX009 treatment, if there is a robust correlation between the target expression and the pharmacology efficacy. It is worth noting that PD-L1 IHC companion diagnostics (CDx) are commercially available and are commonplace in the clinics for PD1 and PD-L1 treatments.

**Table 2:** Expression levels of CD47 and PD-L1 immune checkpoints in B-lymphoma PDXs as well as models’ sensitivity to HX009.

ID	mRNA-CD47^#^	H-Score-CD47	mRNA-PD-L1^#^	H-Score-PD-L1	TGI%	Comments
LY2345	6.5	265	4.0	175	48	
LY6701	5.8	101	− 0.4	53	9	
LY12966	5.9	175	− 1.1	0	19	
LY12962	5.7	163	− 1.7	0	51	
LY2266	6.6	129	5.9	190	41	High PD-L1^+^TIL*
LY2264	6.5	49	5.0	0	6	High PD-L1^+^TIL*
LY6443	6.7	260			32	

IHC scores is based on tumor cell staining; *is based on non-tumor leukocyte staining.

^#^The mRNA levels are represented in log2(FPKM) based on RNAseq dataset. FPKM: fragments per kilobase per million mapped reads. The lowest log2(FPKM) value as − 2. %: Tumor growth inhibition.

### CD47^hi^ PDXs seemed to be more sensitive to HX009

Our working hypothesis was that the expression levels of ICs predict HX009 efficacy in lymphoma. We tested this hypothesis by employing this same small cohort of lymphoma PDXs (Table 2) by pharmacologic-modeling HX009 in a “mouse clinical trial format”^46,47^. Due to the immunodeficient nature of PDX models, only CD47 targeting could be assessed as above xenograft modeling. The results are summarized in Table 2 and a representative tumor growth inhibition curves was shown in Fig. 3D and Supplement Fig. S3D, both suggest that CD47^hi^ models may represent better responders to HX009, or CD47 expression could be a positive predicator for HX009 pharmacology. Although we could not demonstrate the influence of PD-L1/L2 levels on HX009 pharmacology in these PDX models due to their immune-deficiency, it may be reasonable to speculate that the high expression of PD-L1/L2 could potentially also be positive predictors for HX009 pharmacology in human. Due to the co-regulation described above (Table 2 and Fig. 3D, Supplement Fig. S3D), the dual targeting HX009 should likely be more effective than the respective single-targeting in human lymphoma patients.

## Discussion

The blockade of PD1/PDL1 has been recognized as a significant incremental step in the successful treatment of solid tumors, though is often associated with far fewer compelling results in other hematologic malignancies. Combination therapies that integrate checkpoint blockade with chemotherapy or other biologics, including other checkpoint inhibitors, *e.g.* CTLA4 mAb, appear to be emerging as the next promising step in the development of this important class of drugs. The preclinical data presented herein appears to support the contention that combinations of checkpoint targeted drugs, in this case the dual targeting of PD1 and CD47, may be associated with improved activity in models of lymphoma not historically recognized to be sensitive to PD1 or CD47 blockade.

The first generation CD47-targeting agents, exemplified by CD47 mAbs^8,50^ and SIRPα fusion proteins^21,26^, are being tested in the patients, mostly in hematopoietic malignances. They generally exhibited only modest activity across the broader spectrum of these diseases^8,23^. Most of these trials have evolved to some combination strategy as the means to improve on the activity seen with the monotherapy targeting CD47^8,23,26^. Emerging literature suggests rational combinations, particularly with other ICIs, likely to enhance efficacy because of the dual targeting of both innate and adaptive checkpoints^13,14,27,29^. These clinical and preclinical experiences again support the rationale of dual/multi-targeting, such as we have shown with HX009. In addition, many of the early CD47 targeting agents appear to be IgG1 based, which may create major safety challenges as described above, which may not be mitigated by simple combination strategies^13,29,51^. The rational engineering of specifically targeted bispecific antibodies may represent an opportunity to improve activity, while reducing toxicity.

Given HX009 itself represents a combination therapy within the single antibody design, our expectation is that this feature alone will improve on the activity of traditional monotherapies directed against these targets by fulfilling the theoretical benefits of targeting both the innate and adaptive immune system^13,14^. The characterization of the biophysical properties of HX009 in fact confirmed that the molecule exhibits all the pre-determined features as envisioned at the outset. Importantly these represent potent binding to PD1, with consequent functional effects similar to what one would expect with a traditional anti-PD1 directed mAb. Additionally, the CD47 targeted Fc-SIRPα component exhibited reduced affinity compared to the existing CD47 directed monotherapies by fusing to the C-terminus of heavy chain (Fc) of a clinically well vetted PD1 antibody molecule and reduce CD47 binding, thereby reducing binding to erythrocytes and megakaryocytes^29,52^. This combination of effects appears to have translated successfully in the humanized mouse model, which in fact established a complimentary effect of the dual targeted HX009^8,21,50,52^. The following two additional considerations of molecular design/engineering have been adopted in order to further improve the safety and pharmacologic profiles of HX009: (1) a PD1 antibody was used, instead of PD-L1 antibody, for two reasons. PD1 antibody has tendency to target high PD1 expressing tumors (TME), limiting interactions with non-malignant peripheral tissues^30^. While PD-L1 targeting could be an obvious component of a BsAb^13,27^ alterative to PD1 targeting, PD-L1 antibody is often associated with the “antigen sink” effect due to the broad expression found on normal tissues. In addition, PD-L1 antibody cannot block PD-L2 ligand, while PD1 antibody can. (2) IgG4, instead of IgG1, was chosen as the antibody backbone in order to reduce the toxicity.

The current paradigm regarding MOAs of CD47 targeting for cancer therapy is largely based on the tumor cell bearing CD47 targeting to block its binding to SIPRα ligand on macrophages and DCs^13,14^, which activate both anti-tumor innate and adaptive immunity. However, recent observations increasingly pointed to the role of CD47 targeting on TME, *e.g*. T_eff_ cells^37,53–56^. With regard to the potential CD47 targeting MOAs of HX009, the anti-lymphoma effect could be attributed to targeting to CD47 either on tumor cells or on T_eff_ CD47 (or even other immune cells in TME), or on both. We have confirmed anti-lymphoma activity of the tumor cell CD47 targeting as shown in xenograft modeling described in this report, although we have yet to demonstrate direct evidence of the role of T_eff_ CD47 targeting in anti-lymphoma activity. However, in theory, HX009 has unique design particularly efficient dual target both PD1 and CD47 on the same cells due to high binding avidity, where PD1 engagement is the initial driving binding and results in subsequent efficient CD47 high avidity binding even with low affinity. Our Jurkat-T-cell reporter assay in vitro consistently demonstrated higher potency of HX009 over HX008 (PD1-mAb) (Fig. 2A), which suggested that anti-CD47 function of HX009 could indeed enhance PD1 mediated T-cell activation. Nevertheless, it would still be important to further investigate the relative contribution of potential targeting T_eff_ CD47 MOAs^37^. Further HuGEMM experimentations using unmodified syngeneic cell, A20, for instance, can be used to confirm the role of T_eff_ (or other TILs) CD47 targeting in HX009 pharmacology.

The data collected to date support the clinical development of HX009 for the treatment of different types of lymphoma. Given the reduced toxicity (unpublished) appreciated in the animal models, HX009 is unlikely to require priming doses which has been necessary with some of the first generation CD47 targeting agents^8,23^. It is also probably that combinations with other standard of care treatment such as rituximab could further enhance the efficacy of HX009 in the treatment of B-cell malignancies, similar as several early CD47-mAb therapies (NCT02663518, NCT03530683, NCT03717103, NCT02367196). Since many early clinical trials based on PD1 and CD47 targeting have not yet produced compelling results for the overwhelming majority of hematological malignances, it will be of interest to compare and contrast the merits of this bispecific approach over the single targeted directed antibodies studied to date. Presently, HX009 is undergoing extensive clinical study in patients with a variety of different types of NHLs (ClinicalTrials.gov Identifier: NCT05189093)^57^.

Finally, it is important to appreciate why certain tumors appear to respond to anti-PD1 and anti-CD47 directed therapies, and why others do not. The differences lie in the tumor cell proper, TME or the competency of the host immune response. Appreciating these differences, as might be revealed through a particular biomarker, could help tailor the use of these drugs in the future, and even inform the best combination partners. Our preclinical PDX trial on a small cohort of lymphoma models under immune deficiency seemed to inform that CD47^hi^ should be a positive predictor for HX009 efficacy. Since CD47 seemingly co-regulated with PD-L1/L2 (Fig. 3 and Table 2), these CD47^hi^ also express higher levels of PD-L1/L2 and should have additional efficacy contribution from anti-PD1 function of HX009 in patients in theory. In another word, companion diagnostics (CDx) based on the expression of these two ICs could be useful in guiding the future patient trials. There has already been marketed CDx based on IHC for PD-L1. CDx for CD47 based on the IHC can readily be developed as well. Since most readily accessible clinical samples of lymphoma is the FFPE, it is reasonable to develop and employ IHC based CDx for HX009 clinical development.

## Materials and methods

### Cell cultures and reagents

Raji, Jurkat A20 and Karpas-299 cells, from American Type Culture Collection (ATCC), were cultured in RPMI-1640 (Hyclone) with 10% FBS (Gibco). 293T-PD1 cell stably transfected of PD1 lentiviral vector was created in our laboratory by using 293T cells from ATCC and cultured in high glucose DMEM medium (Hyclone) with 10% FBS (Gibco) and puromycin. CHO-PDL1-CD3L and Jurkat-PD1-NFAT cell lines were obtained from National Institutes for Food and Drug Control (China), cultured in DMEM/F12 (Gibco) or RPMI-1640 (Gibco) medium containing 10% FBS, (Gibco), 1% NEAA (Gibco). Jurkat-CSR cells, from ImmuneOnco Biopharmaceuticals (Shanghai), were cultured in RPMI-1640 (Gibco) with 10% FBS (Gemini). Human PBMCs used in the studies were purchased from ALLCELL (https://allcells.com) where informed consent and approved procedures by its institution committee were used for sample collection and preparations. HX009, HX008, Magrolimab and hSirpα-Fc were constructed in-house in our laboratory. Magrolimab were constructed strictly according to the provided sequence from IMGT database (https://www.imgt.org/3Dstructure-DB/).

### Target protein binding/ligand blocking assays based on ELISA

PD1-his or CD47-his recombinant protein (Genescript) was coated in 96-well plates at 4^0^C overnight. Serial dilutions of HX009 or other proteins were applied to duplicate wells for 1 h. The bound antibodies were detected by horseradish peroxidase (HRP)-conjugated goat anti-mouse antibodies (Jackson ImmunoResearch) and developed with 3,3′5,5′-tetramethylbenzidine (TIMB, Biosharp) substrates. Ligand blocking assay ELISA was performed according to Zhang et al.^35^ with minor modifications. Briefly, the IC_50_ (half maximal inhibitory concentration) of HX009 competing with the PD-L1 to bind to PD1 was determined by ELISA by serially diluting HX009 (9 × dilution gradient) into binding samples containing defined amount of PDL-1-mFc or CD47-mFc. The binding EC_50_ of HX009 to PD1 protein or binding blocking were determined by fitting the dose–response data to a four-parameter simulation model.

### Binding to target receptor^+^ cell surface by flow cytometry

PD1 stably transfected 293T cell was used to determine the PD1^+^ cell binding of a serially diluted HX009 samples using flow cytometry using CytoFLEX flow cytometer (Beckman). Jurkat and Raji cells were used for CD47^+^ cell binding of a serially diluted HX009 similarly.

### Reporter assays for functional interaction with PD1 and CD47

For assaying biological PD1 blocking, Jurkat-PD1 cells carrying an IL-2 promoter-guided luciferase reporter gene were in vitro cocultured with precoated CHO-PD-L1-CD3L cells expressing TCR stimulatory molecules and PD-L1, with addition of HX008 and HX009. The luciferase readout was detected by measuring chemiluminescence at 560 nM wave length using Bio-Lite™ Luciferase Assay System (Vazyme, Najing). Dose–response curve was fitted using four-parameter simulation equation.

For assay CD47 blocking, Jurkat-CSR cell were exposed to CD47-hFc (50 ng/mL), with the presence or absence of HX009 of serially diluted HX009, controlled by hSIRPα-Fc or Magrolimab. The cell viability was analyzed by CCK-8 reagent (Dojindo) after 48 h incubation. The experimental procedures follow the instructions from the manufacturer. The signal was obtained according to absorbance at 450 nm wave length (OD_450_). Cell death index (CDI) was calculated as CDI (%) = (1 − (OD_450 sample_ − OD_450 blank_)/(OD_450 positive control_ − OD_450 blank_))*100%.

### Reactivation of SEB stimulated PBMC and mixed lymphocyte reaction (MLR) assays

Human PBMC were isolated from healthy donors using Ficoll density gradient separation. After a 4-h incubation at 37 °C, suspended T cells were taken and cultured in RPMI medium containing 10% FBS. T cells were treated with 50 ng/mL of SEB (Beijing Compro Benin) and then incubated with serial dilution of HX009 and HX008 for 3 days. Secretion levels of IFN-γ and IL-2 were analyzed using a commercial ELISA kit with the manufacturer’s instructions (Dakewei). The blank and only SEB treatment were also assessed to indicate the basal cytokine secretion level.

MLR assay was assessed according to protocol presented in Zhang et al.^35^ with minor modifications. Briefly, human PBMC were isolated from healthy donors using ficoll density gradient separation and monocytes were isolated using a CD14 positive selection kit (Miltenyi). Dendritic cell maturation was accomplished with cytokine cocktail treatment. Matured dendritic cells and T cells from another unrelated individual were mixed and incubated with serial dilution of HX009 and HX008. The effect of HX009 was indirectly evaluated by detecting the secretion levels of IFN-γ and IL-2 using commercially available ELISA kits in accordance with the manufacturer’s instructions (Dakewei). The data is statistical (t-test) analyzed between HX009 and isotype control (hIgG4) to determine HX009 dose-dependent-stimulation of the IFN-γ and IL-2 cytokine secretion (P < 0.01 or P < 0.05).

### In vivo mouse anti-tumor pharmacology modelling

All in vivo murine experiments were conducted under sterile conditions at Crown Bioscience SPF facility in strict accordance with the Guide for the Care and Use of Laboratory Animals of the National Institutes of Health. The protocol was approved by the Committee on the Ethics of Animal Experiments of Crown Bioscience (Crown Bioscience IACUC Committee). The study design all followed the ARRIVE Guideline. The antitumor treatment studies were conducted by following protocols described in detail elsewhere^40,58^ with certain modifications. For the Raji cell derived B-lymphoma xenograft model study, the tumor cells were subcutaneously implanted into NOD/SCID mice (GemPharmtech) whose polymorphic SIRPα efficiently interact with human CD47 proteins on the xenograft tumor cells^38,39^. The treatment groups include HX009 (100 mg/kg, intravenously or IV, twice weekly), SIRPα-Fc (positive control, 8.9 mg/kg, equal molar as HX009) and vehicle. When the average tumor volume reached ~ 100 mm^3^, the mice were randomly grouped and treated. Tumor growth inhibition (TGI) was calculated as TGI% = (1 − V_treatment_/V_control_) × 100.

For Karpas-299 MiXeno T-lymphoma model, Karpas-299 cells were transplanted subcutaneously followed by human PBMC (IV) engraftment four days later^40^ in NPG mice (VitalStar, Beijing). Tumor volume was monitored as described in the Raji experiment above, and the reconstitution of human immune cells were monitored by peripheral blood cell flow cytometry^40,59^.

Humanized syngeneic mouse A20-huCD47 B-lymphoma HuGEMM^31,40^ model was constructed by knock-in (KI) of the huCD47 gene into a mouse syngeneic B-lymphoma A20 cell line and four human genes, hPD1, hPDL1, hCD47, hSIRPα, were knocked-in Balb/c mice (HuGEMM™), similarly to that reported elsewhere^44^ (Supplement Fig. S2). Syngeneic A20-hCD47 cells were subcutaneously transplanted as similarly as above xenografts and the pharmacology evaluation of HX009 (10 mg/kg, IP), along with equal molar hSIRPα-Fc (8.8 mg/kg) and HX008 (8.6 mg/kg), in the similar described format.

DLBCL-PDX establishment and treatment pharmacology are described elsewhere, similar to the above xenograft pharmacology, except that tumor fragments are used for implantation widely described before^40^. The original PDX establishment followed the procedure approved by Crown’s relevant committees and the original lymphoma samples were obtained from collaborating hospitals whose institutional committee approved the sample collection and obtained informed consents.

### CD47 and PD-L1 IHC assays of PDX samples

Tissue microarray (TMA) or FFPE slides of both lymphoma PDX models were prepared following standard histology procedure. A standard IHC staining protocol was performed in Bond Rx Automated IHC/ISH Slide Staining machine (Leica Biosystems) using commercial anti-CD47 antibody, clone EPR21794 (Abcam, ab218810) and anti-PD-L1 antibody, clone E1L3N (Cell Signaling Technology, 13684). These staining were followed by whole slide imaging using the NanoZoomer NDP2.0-HT Digital Slide System (Hamamatsu) and quantified and calculated H-Score (see below) using HALO® image analysis software (Indica Labs) with pathologists' peer review. The necrotic tumor regions and blank space in the tissue were excluded and the intensity of positive staining on tumor cells was scored at four levels, 0 (negative), 1 + (weak staining), 2 + (medium staining), 3 + (strong staining). The percentages of tumor cells at different intensity levels were evaluated with formula as:

$$ H - Score = \left( {\% \;at\;0} \right) \times 0 + \left( {\% \;at\;1} \right) \times 1 + \left( {\% \;at\;2} \right) \times 2 + \left( {\% \;at\;3} \right) \times 3\,\left( {H - Score\;range\;is\;0\;to\,300} \right). $$

## Supplementary Information

Supplementary Figures.

## Data Availability

The datasets generated and/or analysed during the current study are not publicly available due to that they are yet to be organized for access, but are available from the corresponding author on reasonable request.
