# Supplementary material for: HX009, a novel BsAb dual targeting PD1 x CD47, demonstrates potent anti-lymphoma activity in preclinical models
Source: Sci Rep. 2023 Apr 3;13:5419. doi: 10.1038/s41598-023-32547-y (PMC10070465; doi:10.1038/s41598-023-32547-y)
Supplement: Supplementary file 1 — Supplementary Figures. [file 41598_2023_32547_MOESM1_ESM.pptx]

## Slide 1
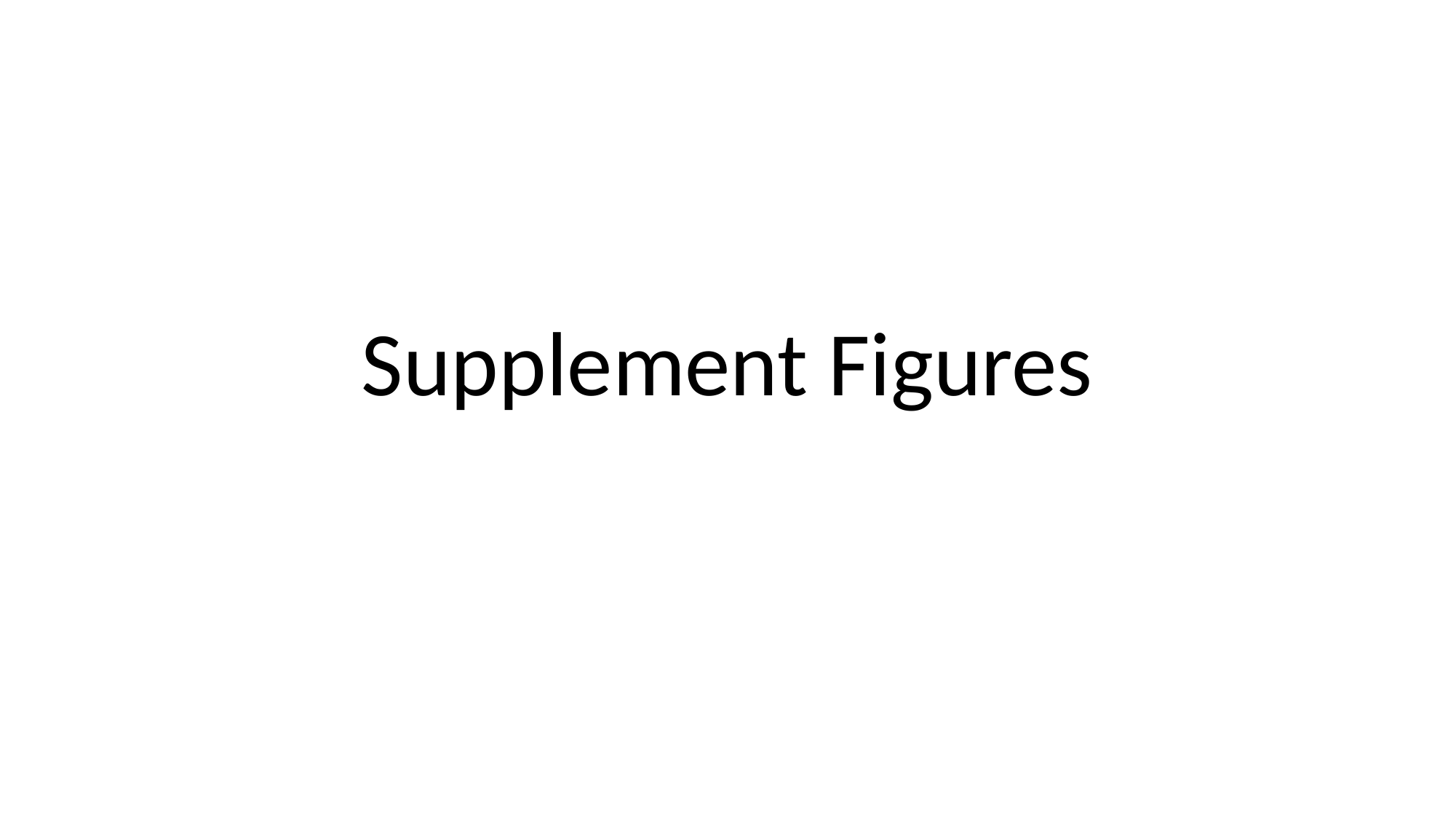

# Supplement Figures

## Slide 2
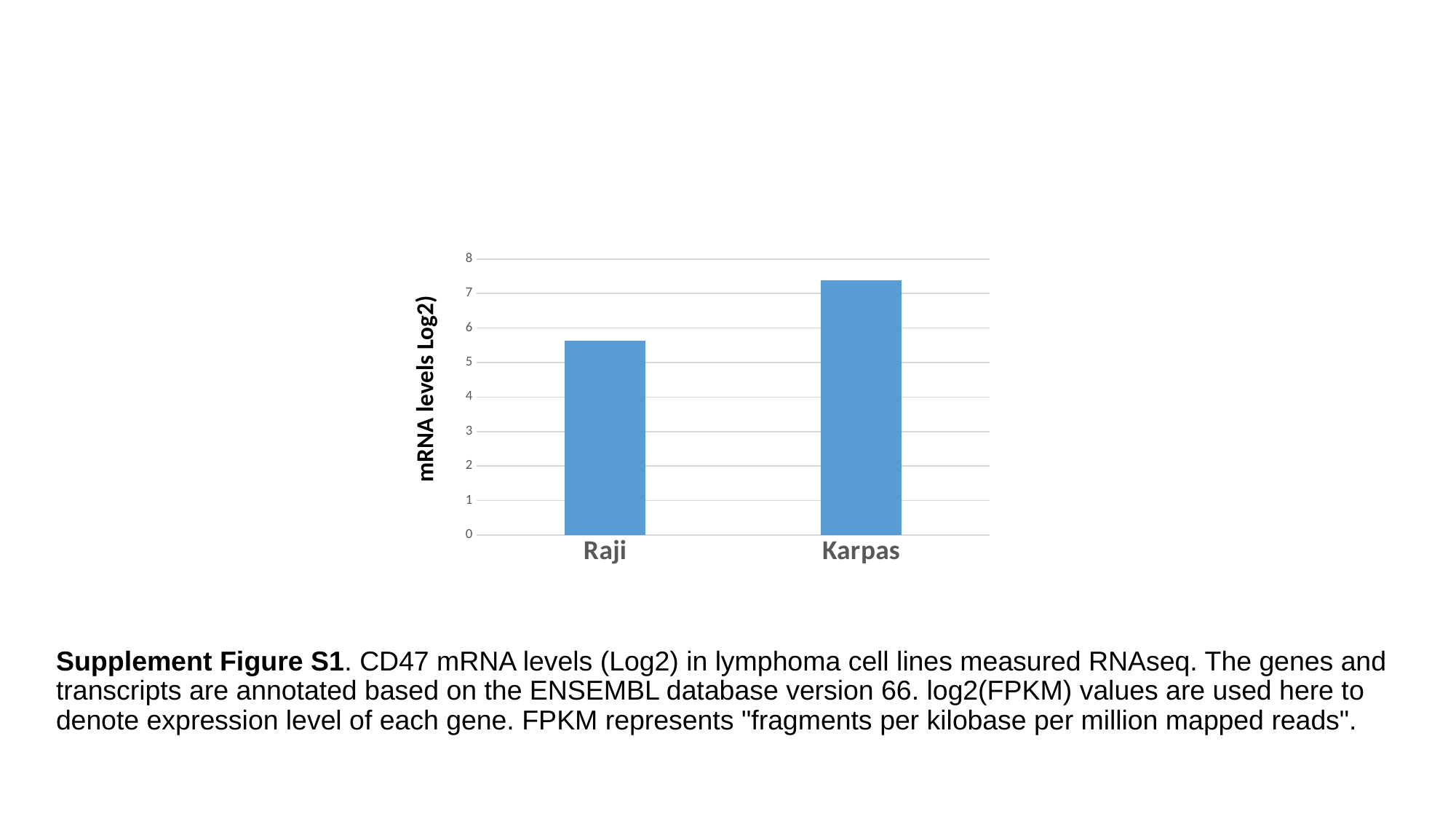

### Chart
| Category | Log2 |
|---|---|
| Raji | 5.6334 |
| Karpas | 7.3829 |mRNA levels Log2)
# Supplement Figure S1. CD47 mRNA levels (Log2) in lymphoma cell lines measured RNAseq. The genes and transcripts are annotated based on the ENSEMBL database version 66. log2(FPKM) values are used here to denote expression level of each gene. FPKM represents "fragments per kilobase per million mapped reads".

## Slide 3
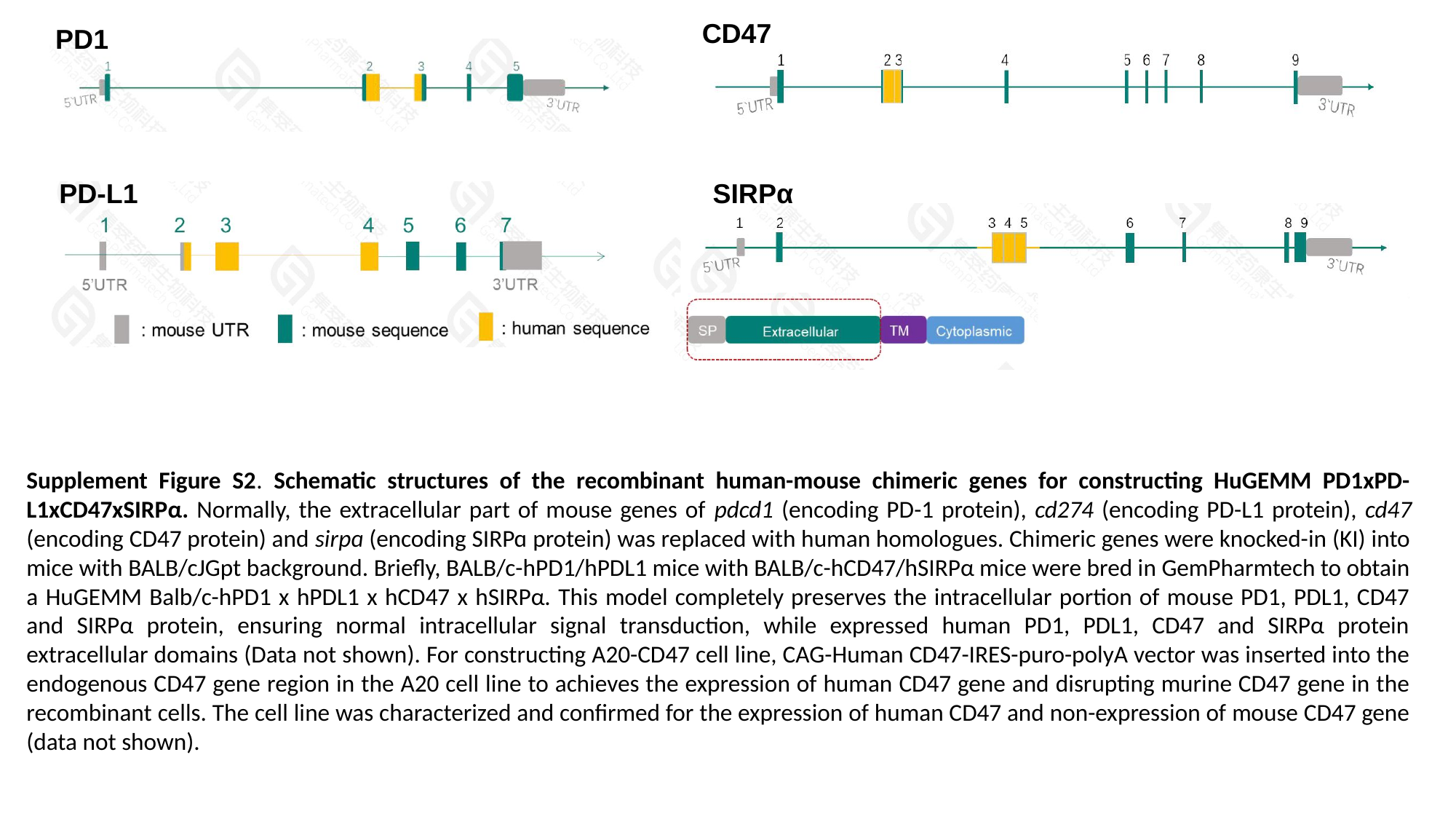

CD47
PD1
PD-L1
SIRPα
Supplement Figure S2. Schematic structures of the recombinant human-mouse chimeric genes for constructing HuGEMM PD1xPD-L1xCD47xSIRPα. Normally, the extracellular part of mouse genes of pdcd1 (encoding PD-1 protein), cd274 (encoding PD-L1 protein), cd47 (encoding CD47 protein) and sirpɑ (encoding SIRPɑ protein) was replaced with human homologues. Chimeric genes were knocked-in (KI) into mice with BALB/cJGpt background. Briefly, BALB/c-hPD1/hPDL1 mice with BALB/c-hCD47/hSIRPα mice were bred in GemPharmtech to obtain a HuGEMM Balb/c-hPD1 x hPDL1 x hCD47 x hSIRPα. This model completely preserves the intracellular portion of mouse PD1, PDL1, CD47 and SIRPα protein, ensuring normal intracellular signal transduction, while expressed human PD1, PDL1, CD47 and SIRPα protein extracellular domains (Data not shown). For constructing A20-CD47 cell line, CAG-Human CD47-IRES-puro-polyA vector was inserted into the endogenous CD47 gene region in the A20 cell line to achieves the expression of human CD47 gene and disrupting murine CD47 gene in the recombinant cells. The cell line was characterized and confirmed for the expression of human CD47 and non-expression of mouse CD47 gene (data not shown).

## Slide 4
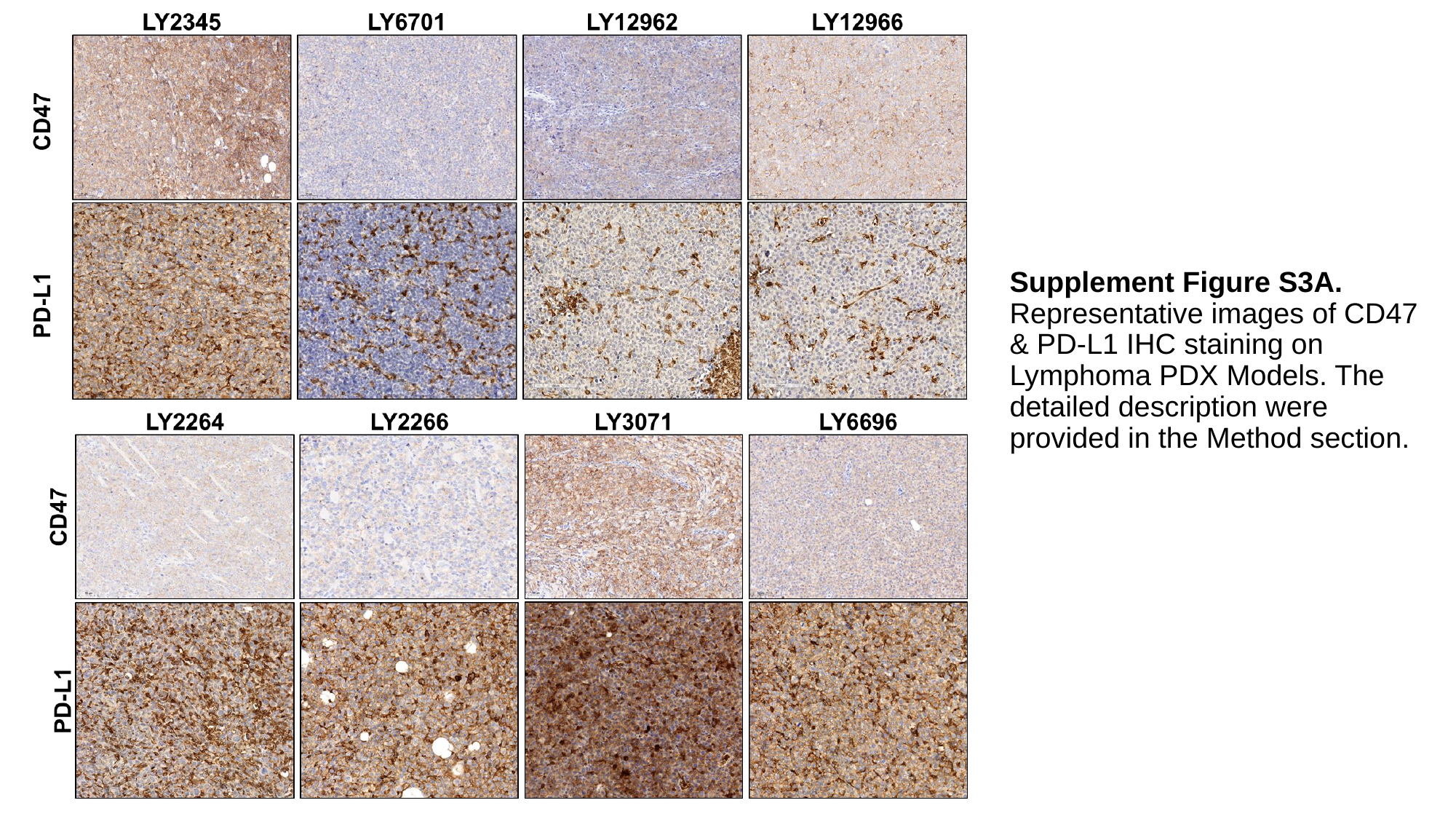

Supplement Figure S3A. Representative images of CD47 & PD-L1 IHC staining on Lymphoma PDX Models. The detailed description were provided in the Method section.

## Slide 5
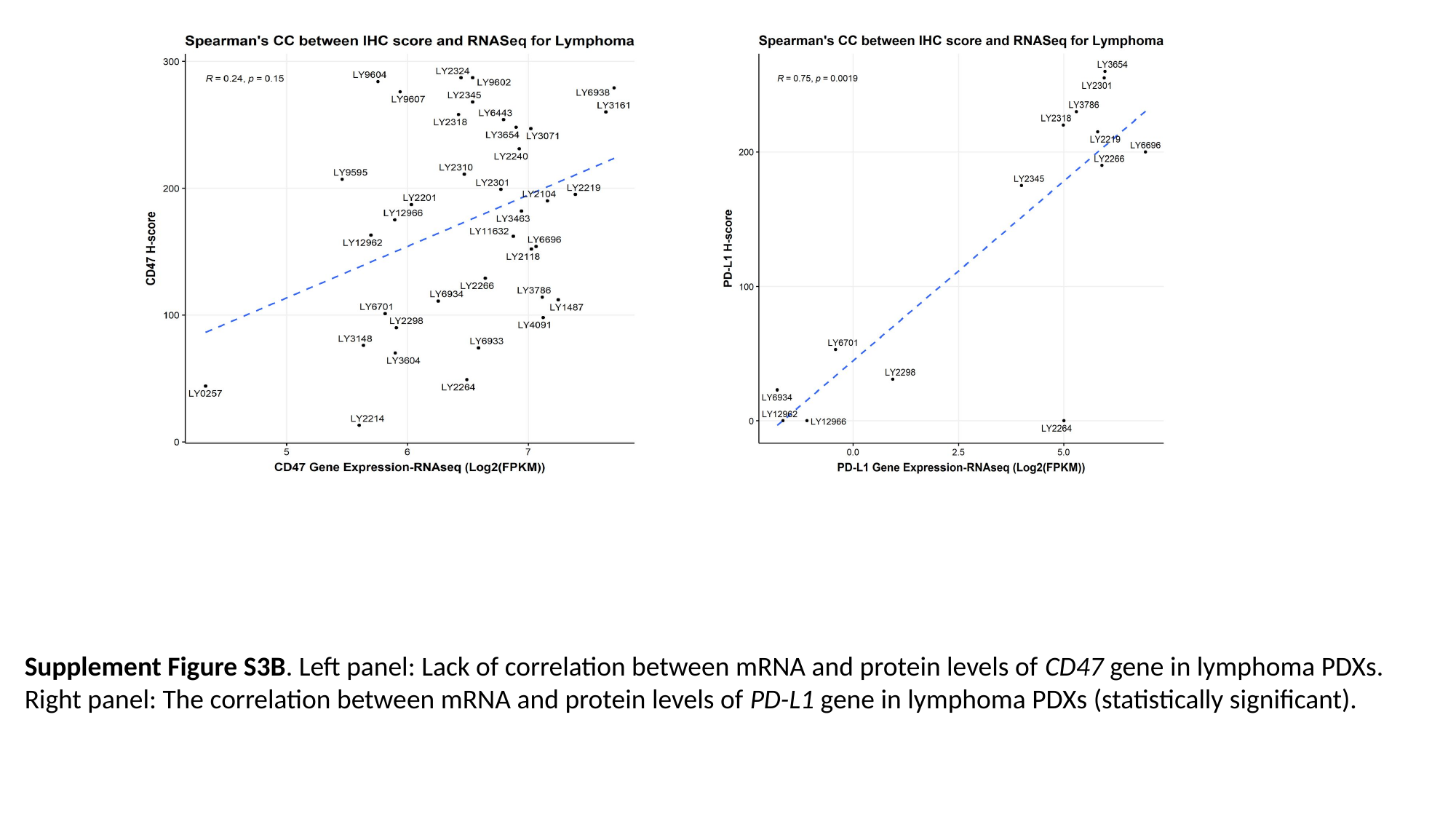

Supplement Figure S3B. Left panel: Lack of correlation between mRNA and protein levels of CD47 gene in lymphoma PDXs. Right panel: The correlation between mRNA and protein levels of PD-L1 gene in lymphoma PDXs (statistically significant).

## Slide 6
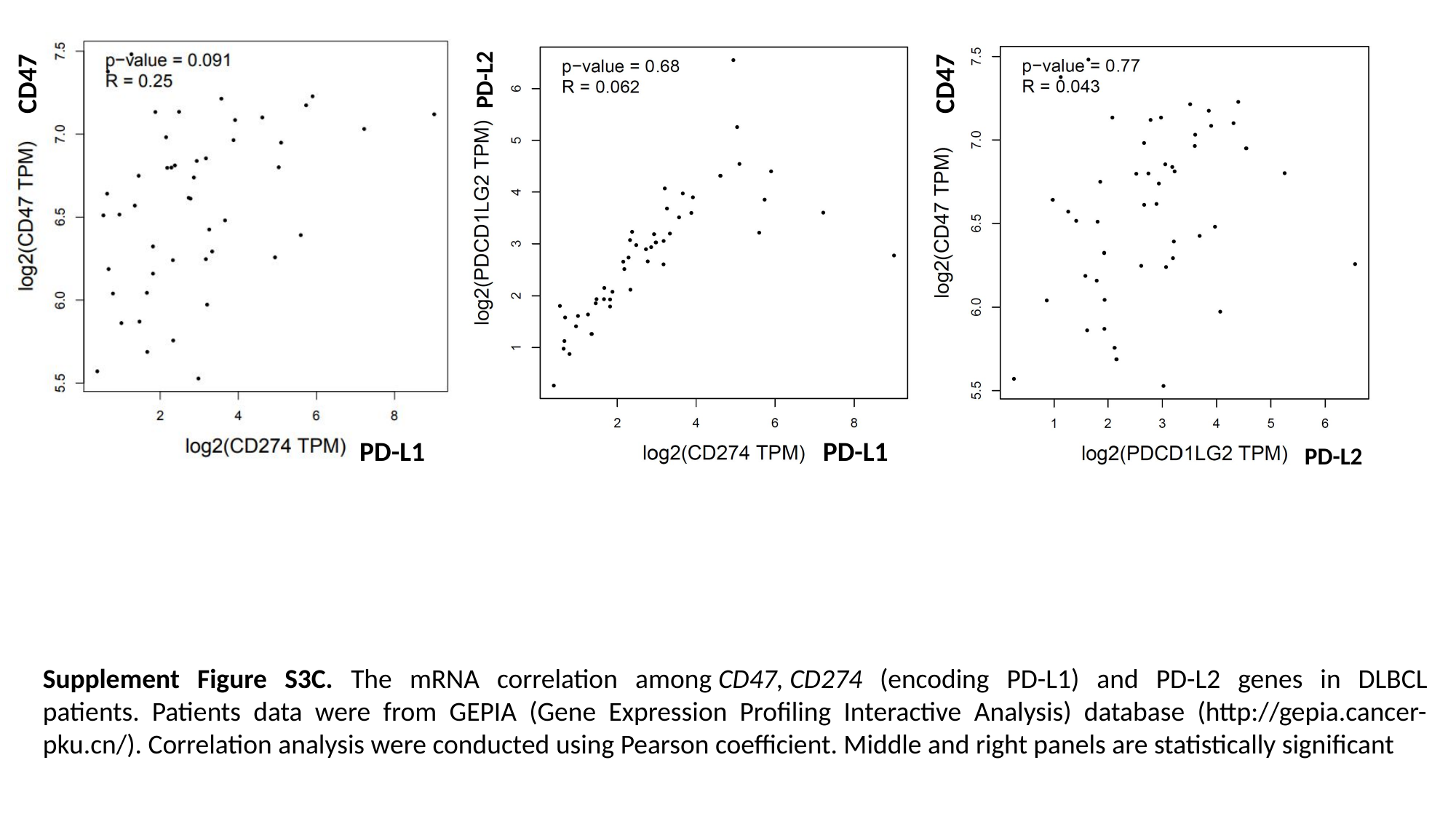

PD-L2
CD47
CD47
PD-L1
PD-L1
PD-L2
Supplement Figure S3C. The mRNA correlation among CD47, CD274 (encoding PD-L1) and PD-L2 genes in DLBCL patients.  Patients data were from GEPIA (Gene Expression Profiling Interactive Analysis) database (http://gepia.cancer-pku.cn/). Correlation analysis were conducted using Pearson coefficient. Middle and right panels are statistically significant

## Slide 7
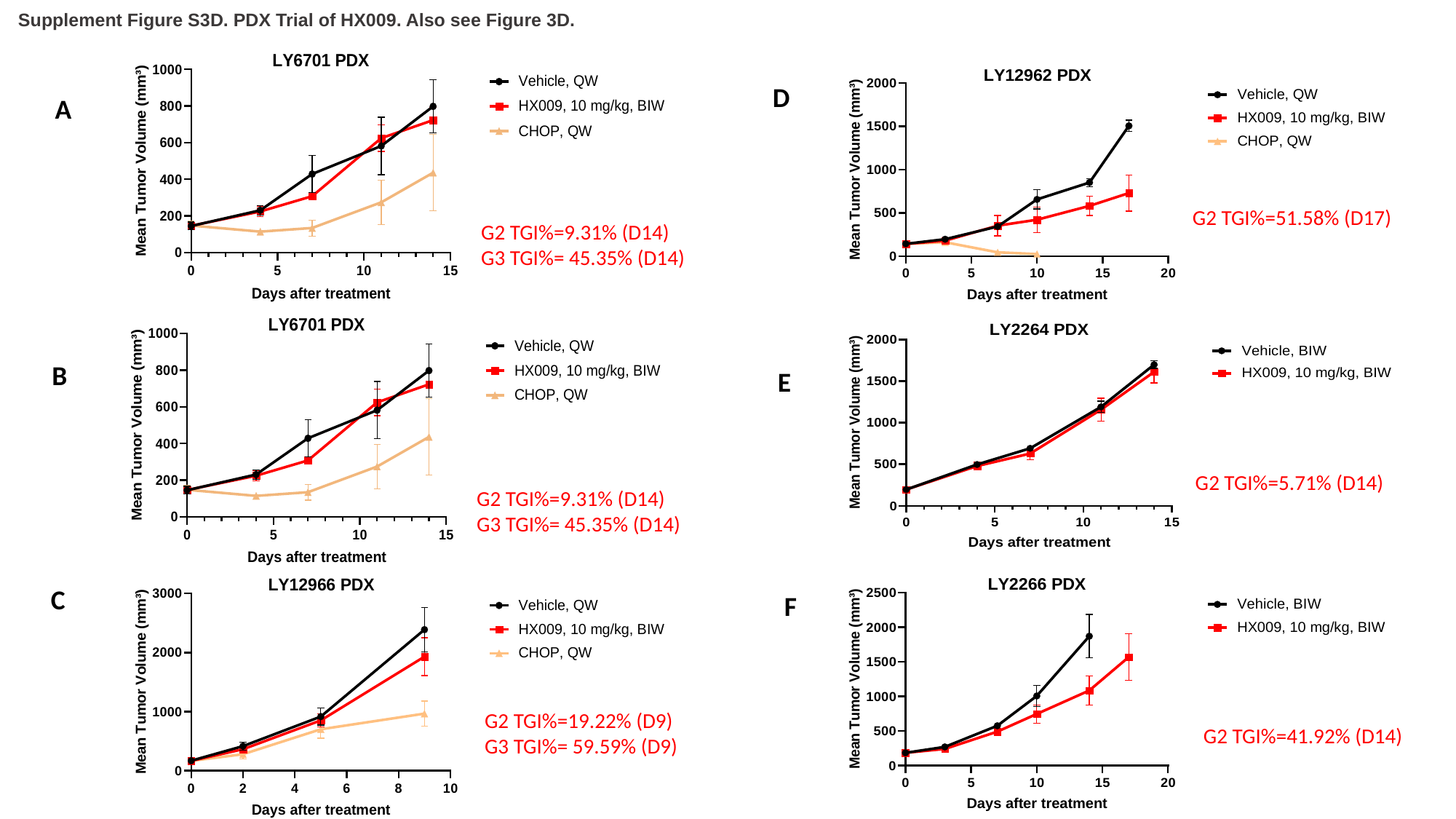

Supplement Figure S3D. PDX Trial of HX009. Also see Figure 3D.
D
A
G2 TGI%=51.58% (D17)
G2 TGI%=9.31% (D14)
G3 TGI%= 45.35% (D14)
B
E
G2 TGI%=5.71% (D14)
G2 TGI%=9.31% (D14)
G3 TGI%= 45.35% (D14)
C
F
G2 TGI%=19.22% (D9)
G3 TGI%= 59.59% (D9)
G2 TGI%=41.92% (D14)
